# Poly-GR dipeptide repeat polymers correlate with neurodegeneration and Clinicopathological subtypes in *C9ORF72*-related brain disease

**DOI:** 10.1186/s40478-018-0564-7

**Published:** 2018-07-20

**Authors:** Nobutaka Sakae, Kevin F. Bieniek, Yong-Jie Zhang, Kelly Ross, Tania F. Gendron, Melissa E. Murray, Rosa Rademakers, Leonard Petrucelli, Dennis W. Dickson

**Affiliations:** Department of Neuroscience, Mayo Clinic, 4500 San Pablo Road, Jacksonville, FL 32224 USA

**Keywords:** C9ORF72, Dipeptide repeat polymers (DPR), Poly-GR, Neurodegeneration, Dimethylarginine

## Abstract

Frontotemporal lobar degeneration (FTLD) is heterogeneous in clinical presentation, neuropathological characteristics and genetics. An expanded GGGGCC hexanucleotide repeat in *C9ORF72* is the most common genetic cause of both FTLD and motor neuron disease (MND). Dipeptide repeat polymers (DPR) are generated through repeat-associated non-ATG translation, and they aggregate in neuronal inclusions with a distribution distinct from that of TDP-43 pathology. Recent studies from animal and cell culture models suggest that DPR might be toxic, but that toxicity may differ for specific DPR. Arginine containing DPR (poly-GR and poly-PR) have the greatest toxicity and are less frequent than other DPR (poly-GP, poly-GA). A unique feature of arginine-containing DPR is their potential for post-translational modification by methyl-transferases, which produces methylarginine DPR. In this report, we explored the relationship of DPR and methylarginine to markers of neurodegeneration using quantitative digital microscopic methods in 40 patients with *C9ORF72* mutations and one of three different clinicopathologic phenotypes, FTLD, FTLD-MND or MND. We find that density and distribution of poly-GR inclusions are different from poly-GA and poly-GP inclusions. We also demonstrate colocalization of poly-GR with asymmetrical dimethylarginine (aDMA) immunoreactivity in regions with neurodegeneration. Differences in aDMA were also noted by clinical phenotype. FTLD-MND had the highest burden of poly-GR pathology compared to FTLD and MND, while FTLD-MND had higher burden of aDMA than FTLD. The results suggest that poly-GR pathology is associated with toxicity and neurodegeneration. It remains to be determined if dimethylarginine modification of poly-GR could contribute to its toxicity.

**Electronic supplementary material:**

The online version of this article (10.1186/s40478-018-0564-7) contains supplementary material, which is available to authorized users.

## Introduction

Frontotemporal lobar degeneration (FTLD) is heterogeneous in clinical presentation, neuropathological characteristics and underlying genetic causes. Clinically, FTLD manifests in changes in behavior, personality and language, but it can also be associated with motor neuron disease (MND), including amyotrophic lateral sclerosis (upper and lower motor neurons affected), progressive muscular atrophy (lower motor neurons predominantly affected) and primary lateral sclerosis (upper motor neurons primarily affected) [8, 12, 13]. Hexanucleotide repeat expansions in chromosome 9 open reading frame 72 gene (*C9ORF72*) are the most common genetic cause of FTLD and MND [7, 24], which are collectively referred to as c9FTLD-MND. The neuropathological features of c9FTLD-MND include nuclear RNA foci, inclusions composed of repeat-associated non-ATG (RAN) translated dipeptide repeat polymers (DPR) and TDP-43 pathology. Of these features, DPR pathology and nuclear RNA foci are unique and highly specific to c9FTLD-MND. RAN translation can occur in both sense and antisense directions generating five different DPR: poly-(glycine-alanine/poly-GA), poly-(glycine-arginine/poly-GR) and poly-(glycine-proline/poly-GP) encoded by the sense strand, and poly-(proline-alanine/poly-PA), poly-(proline-arginine/poly-PR) and poly-(proline-glycine/poly-PG) by the antisense strand [1, 11, 21]. In human brains, DPR inclusions are detected predominantly in neurons as cytoplasmic inclusions, but nuclear inclusions and rare inclusions in glial cells are also found [26]. There is evidence that DPR inclusions are detected in presymptomatic individuals before significant neurodegeneration and TDP-43 pathology is detected [19, 32]. Recent progress in developing animal models of c9FTLD-MND suggests that toxicity of specific DPR polymers is variable. Several pathogenic mechanisms, not mutually exclusive, may be at play including nuclear dysfunction, altered RNA splicing, impaired nucleocytoplasmic transport, altered RNA granule dynamics, and disruption of proteostasis. Of the various DPR species, poly-PR and poly-GR are most toxic in Drosophila [20] and in cell culture models [9, 15, 30, 31, 33, 34]. The reasons that poly-PR and poly-GR are more toxic remain unknown, but given their high arginine content, one might hypothesize that methylarginine post-translational modification might contribute to their toxicity. Arginine residues in polypeptides can be modified by methyltransferases to conjugate one (monomethylarginine) or two (dimethylarginine) methyl groups. Dimethylarginine modifications have been reported in proteins in human plasma and urine, and their levels are increased in conditions associated with enhanced protein breakdown, such as tumor growth and neurodegenerative disorders [27]. There are two isomers of dimethylarginine, symmetric dimethylarginine (sDMA) and asymmetric dimethylarginine (aDMA). The biologic function of DMA is not well known; however, elevated levels of aDMA in plasma predict poor prognosis in many diseases, such as cerebrovascular disease and Crohn’s disease, where DMA modification is considered toxic [29]. The presence of DMA modifications has not been specifically studied in c9FTLD-MND.

Several studies have reported clinicopathological correlates of DPR in brains of c9FTLD-MND, but most have been relatively small autopsy series and used mostly semiquantitative methods [6, 17, 26]. It remains to be determined if there are correlations of specific DPR with clinical or neuropathological subtypes of *C9ORF72*-related disease. To address this issue, we sought evidence to support our hypothesis that arginine-containing DPR, poly-GR in particular, might correlate with the severity of neuropathology and that DMA modification might be related to a gain of toxicity in poly-GR. To investigate this, we systematically evaluated sense strand DPR (poly-GA, poly-GP and poly-GR), as well as aDMA in 40 patients with FTLD, FTLD-MND or MND. We found that poly-GR pathology correlated with neurodegeneration and clinicopathologic subtype. Further, we detected a correlation between the distribution of poly-GR and aDMA pathologies. Taken together, our results suggest a possible mechanism of poly-GR toxicity that could be the basis of novel therapeutic approaches.

## Material and methods

### Case materials

Forty cases of FTLD or MND with C9ORF72 repeat expansion mutation were obtained from the Mayo Clinic brain bank. The C9ORF72 expansion carriers had pathological diagnoses of FTLD, MND or FTLD-MND. All cases were submitted to or autopsied by the brain bank for neurodegenerative disorders at the Mayo Clinic in Jacksonville, Florida. Clinical information (age at death, sex, clinical diagnosis, disease duration, and family history) was obtained from available medical records. The left hemibrain was fixed in 10% formalin, and the right hemibrain was frozen at − 80 °C. Formalin-fixed tissue was sampled with standardized dissection methods and embedded in paraffin blocks.

### Genetic analyses

All cases had hexanucleotide repeat expansions in *C9ORF72* based on a repeat-primed polymerase chain reaction method that detects expansions of the GGGGCC hexanucleotide, and for most cases, the expansions was confirmed with Southern blotting. [7]

### Diagnostic neuropathology

Gross and macroscopic neuropathological assessment was performed by standardized procedures. Formalin-fixed, paraffin-embedded tissue samples were cut at 5 μm thickness and mounted on glass slides for further study. The neuropathologic assessment included immunohistochemistry for phospho-TDP-43 (pTDP-43; mouse monoclonal; Cosmo Bio USA, Carlsbad, CA) and C9RANT antibody [1]. A TDP-43 pathologic subtype was assigned to each case using the harmonized classification scheme [18] based upon morphology and distribution of lesions in cortical and subcortical regions [14].

### Immunohistochemistry and double-label immunofluorescence

Immunohistochemistry was performed on 5-μm thick sections from formalin-fixed paraffin embedded tissue. Glass-mounted sections were de-parafinized in xylene and rehydrated in ethanol and dH_2_O. Rabbit polyclonal antibodies used for DPR immunohistochemistry were as follows: poly-GA (Rb9880), poly-GP (Rb5823), poly-GR (Rb7810) (Leonard Petrucelli, Mayo Clinic, Jacksonville, FL [1, 11]). A rabbit polyclonal antibody (ASYM24 07–414, MilliporeSigma, Burlington, MA) was used for immunohistochemistry for aDMA. Regions of interest were middle frontal gyrus (FCtx), motor cortex (MCtx) and hippocampus. Adjacent sections were immunostained for phospho-TDP-43 as above. All Immunoperoxidase sections were processed on a DAKO AutostainerPlus (Agilent/DAKO, Santa Clara, CA) with the DAKO EnVisionTM+ system-HRP with diaminobenzidine as the chromogen. Nonspecific antibody binding was blocked with normal goat serum (Sigma, St Louis, MO).

For double immunofluorescence staining, a rat monoclonal antibody was used to detect poly-GR (5H9, MilliporeSigma, Burlington, MA) and a rabbit polyclonal antibody was used to detect aDMA (MilliporeSigma, Burlington, MA). The fluorochromes were Alexa Fluor 568 and Alexa Fluor 488 conjugated to anti-rabbit or anti-rat IgG (Invitrogen/ThermoFisher, Waltham, MA).

### Assessment of neurodegeneration

For assessment of neurodegeneration, H&E stained sections of FCtx, MCtx and hippocampus were evaluated. Degeneration was graded semiquantitatively as absent (0), mild (1), moderate (2) or severe (3) based on the presence of spongiosis, neuronal loss and gliosis. In FCtx, neurodegeneration was typically associated with superficial laminar spongiosis, while in the hippocampal dentate fascia, discontinuity in the density of granular neurons was used as an indicator of neuronal loss and neurodegeneration. In hippocampal subfields – CA4 and CA2/3 – degeneration was assessed by neuronal loss and astrogliosis (see Additional file 1: Figure S1).

### Image analysis

Digital microscopy methods used in this study have been described previously [22]. Briefly, immunostained glass slides were scanned on an Aperio ScanScope XT slide scanner (Aperio Technologies/Leica Biosystems, Buffalo Grove, IL) producing high resolution digital images. The regions of interest in the hippocampus included the entire hippocampus, as well as delimited subregions of dentate fascia, CA4 sector and CA2/3 sector. In MCtx and FCtx, the region of interest was the entire thickness of the cortex where the pial surface was parallel to the cortical gray-white junction. Digital image analysis was performed using Aperio ImageScope software. A color deconvolution algorithm was used to count the number of pixels that were strongly immunostained by the chromogen. The output variable was percentage of strong positive staining relative to total pixels in the region of interest.

Analytic algorithms for poly-GA had to be adapted to exclude signals two standard deviations above and below the mean because some cases had unexplained background staining. Given this limitation, we also used manual counting methods for poly-GA, as well as for aDMA. In all cases, aDMA labeled not only cytoplasmic inclusions, but also many nuclei presumably due to detection of methylated histones or other nuclear proteins [10]. The poly-GA and aDMA manual counts were performed on digital images. In order to make comparisons between the various DPR, it was also necessary to perform manual counts for poly-GP and poly-GR. The number of inclusions detected from manual counts was assessed in the same area traced for image analysis. Manual counts and positive pixel burden from color deconvolution image analysis were highly correlated for both poly-GP and poly-GR (Additional file 2: Figure S2). Only the motor cortex had poor correlation between poly-GP manual counts and positive pixel burden, in part due to prominent cytoplasmic immunoreactivity in large neurons, including Betz cells. These did not represent inclusions in that they had diffuse cytoplasmic staining, instead of compact cytoplasmic inclusion bodies, which were manually counted. As with image analysis, manual counts were expressed as density per square millimeter by dividing the manual count by total area of the annotated region of interest.

### Cell culture experiments

HEK-293 T cells were grown in Opti-Mem plus 10% FBS and 1% penicillin-streptomycin. To examine methylation of poly-GR proteins, HEK-293 T cells were grown in 12-well plates or on coverslips in 24-well plates. Cultures were transfected with 1 μg (12-well plates) or 0.5 μg (24-well plates) of an expression vector[GFP, GFP-(GR)_50_ or GFP-(GR)_100_ using Lipofectamine 2000 (Life Technology). Four hours after transfection, the cultured cells were treated with methylarginine transferase inhibitor, AdOx (A7154, Sigma), at concentration of 5 or 20 μM. DMSO was used as vehicle control. Twenty-four hours after treatment, the cultured cells were harvested for Western blot analysis or fixed for immunofluorescence staining.

### Immunofluorescence staining

HEK-293 T cells were fixed with 4% paraformaldehyde in PBS for 15 min, and then permeabilized with PBS-0.5% Triton X-100 for 10 min. To examine aDMA, cells were blocked with 5% nonfat milk for 1 h at room temperature, and then incubated overnight at 4 °C with rabbit polyclonal anti-aDMA antibody (ASYM24, Millipore). After washing, cells were incubated with the corresponding Alexa Fluor 568-conjugated goat anti-rabbit secondary antibody (1:500, Molecular Probes) at room temperature for 2 h. After washing, they were mounted with Vectashield Mounting Media with DAPI (Vector Laboratories). Images were obtained on a Zeiss LSM 510 META confocal microscope. To quantify the number of cells positive for GFP and aDMA, microscopic fields were randomly selected at 40X magnification. For each field, the number of GFP-positive cells and aDMA-positive aggregates, as well as aggregates with double staining, were manually counted, blinded to condition. These counts were used to calculate the average percentage of aDMA aggregates in cells expressing GFP, GFP-(GR)_50_ or GFP-(GR)_100_.

### Biochemical studies of cell lysates

Cell pellets were lysed in co-immunoprecipitation (co-IP) buffer (50 mM Tris–HCl, pH 7.4, 300 mM NaCl, 1% Triton X-100, 5 mM EDTA) plus 2% SDS, and both protease and phosphatase inhibitors, sonicated on ice, and then centrifuged at 16,000×g for 20 min. Supernatants were saved as cell lysates. The protein concentration of supernatants was determined by BCA assay (Thermo Scientific) prior to Western blot analysis. Cell lysates were diluted with 2 × SDS-loading buffer at a 1:1 ratio (*v*/v), and then heated at 95 °C for 5 min. Equal amounts of protein were loaded into 12-well 4–20% Tris-glycine gels (Novex). After transferring proteins to membranes, membranes were blocked with 5% nonfat dry milk in TBS plus 0.1% Tween 20 (TBST) for 1 h, and then incubated with rabbit polyclonal anti-GFP antibody (A-6455, 1:4000, Life Technologies), rabbit polyclonal anti-GR antibody (Rb7810, 1:2000), rabbit polyclonal anti-aDMA antibody (07–414, 1:1000, EMD Millipore), or mouse monoclonal or GAPDH antibody (H86504M, 1:10000, Meridian Life Science) overnight at 4 °C. Membranes were washed in TBST and incubated with donkey anti-rabbit or anti-mouse IgG antibodies conjugated to horseradish peroxidase (1:5000; Jackson ImmunoResearch) for 1 h. Protein expression was visualized by enhanced chemiluminescence treatment and exposure to film.

### Statistical analyses

Sigma Plot Version 12 (Systat Software, San Jose, CA) was used for statistical analyses. Due to small sample sizes, non-parametric Kruskal-Wallis analysis of variance on ranks (ANOVA on Ranks) was performed on quantitative measures to assess differences between the groups. Post hoc pairwise comparisons were performed between each of the groups using Mann-Whitney rank sum test. For categorical data (e.g., sex and APOE genotype), a Chi-square test was used to compare groups. Fisher’s exact test was used for comparison of pairwise categorical data if the counts were less than 5. Correlative analysis was performed using Spearman rank order correlation. A *p*-value < 0.05 was considered statistically significant.

## Results

### Demographics features of study cohort

The 40 patients with *C9ORF72* mutations included 13 with FTLD, 14 with FTLD-MND and 13 with MND. Demographic information is summarized in Table 1. Patients with FTLD had significantly longer disease duration than patients with MND. They were also significantly older at death compared to both FTLD-MND and MND. The median age at death was 74 for FTLD, 61 for FTLD-MND and 56 for MND. Median disease durations were 6.4 years for FTLD, 3.6 years for FTLD-MND and 2.4 years for MND.

**Table 1:** Demographics of clinicopathologic subgroups

Subgroup	N (F/M)	Age at death	Disease duration
FTLD	13 (2/11)	74 (71, 83)*	6.4 (4.4, 10)*
FTLD-MND	14 (7/7)	61 (60, 68)*	3.6 (2.2, 5.5)
MND	13 (9/4)	56 (50, 68)	2.4 (1.3, 3.5)

All variables analyzed with Kruskal-Wallis ANOVA on Ranks, and data are displayed as median (25th and 75th range), unless otherwise noted

*Statistically significant *p*-value (*p* < 0.05); all p-values for ANOVA on Ranks comparison of all three groups

### Spectrum of poly-GA, poly-GP and poly-GR pathology

**Fig. 1:**
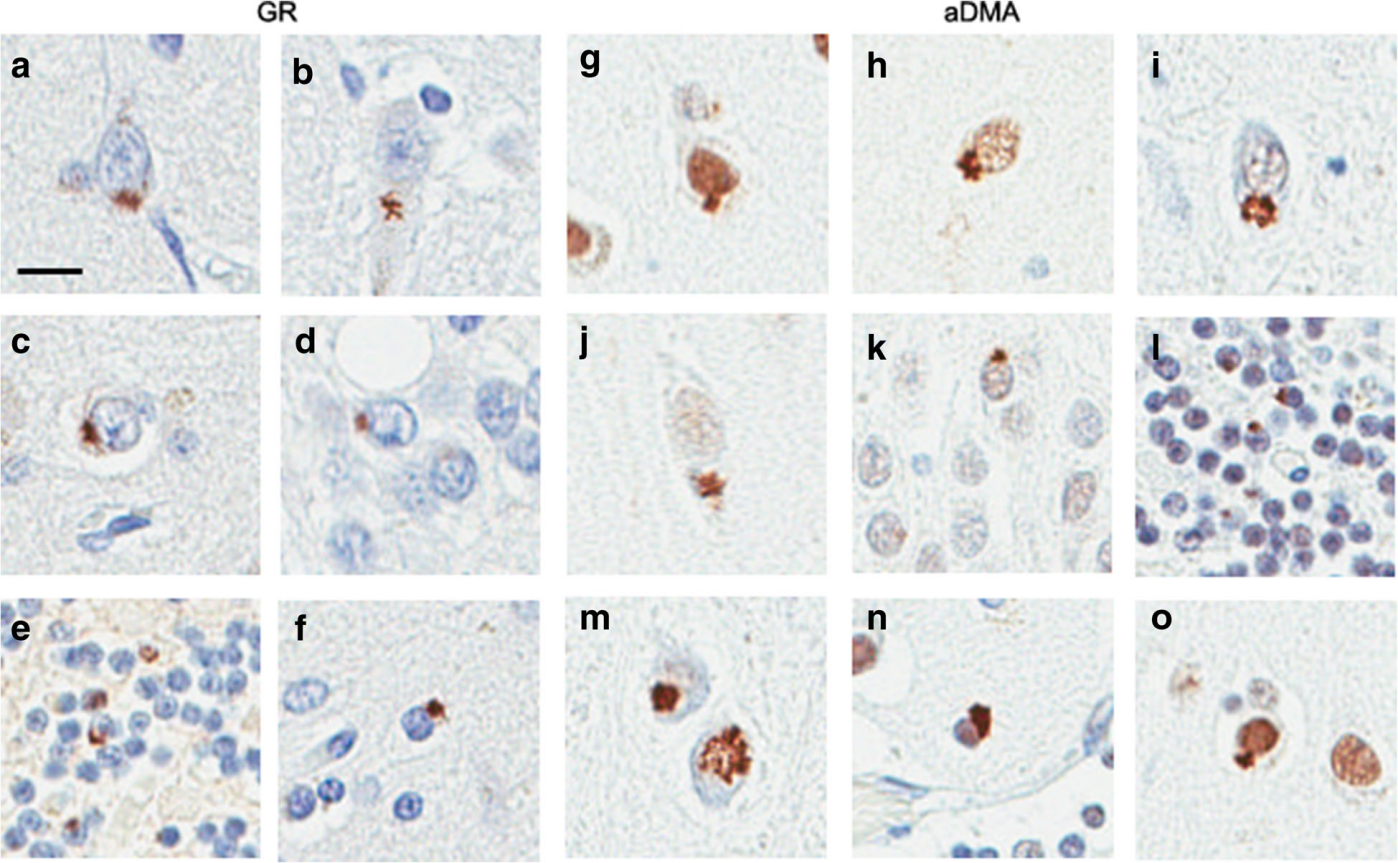
Morphology of poly-GR and aDMA pathology. Immunohistochemistry for poly-GR reveals neuronal cytoplasmic inclusions in frontal cortex (**a**), CA2/3 (**b**), CA4 (**c**), DF (**d**), and cerebellar granular layer (**e**). Inclusions in glial cells were very rare (**f**). Immunohistochemistry for aDMA shows neuronal cytoplasmic inclusions, as well as variable nuclear immunoreactivity, which was dense in some neurons (**g**), moderate in others (**h**) and minimal in others (**i**); inclusions in CA2/3 (**j**), DF (**k**) and (**l**) cerebellar granular layer (**n**); neuronal nuclear inclusions (NII) (**m**); and rare glial cytoplasmic inclusions (**n, o**). Scale bar represents 20 μM. FCtx = frontal cortex, MCtx = Motor cortex, DF = dentate fascia, CA = hippocampal subfields, Cbl = cerebellar granular cell layer

In this study, we limited neuropathological analyses to sense strand DPR (poly-GA, poly-GP and poly-GR) given the paucity of inclusions and the lack of good detection reagents for antisense DPR (poly-PA and poly-PR). We focused particularly on poly-GR pathology, and compared the pathology of poly-GR with poly-GP and poly-GA pathology as these have been shown by both our previous studies and those of others to be the most abundant DPR species in brain samples. Consistent with previous reports, we found that most of the DPR inclusions were neuronal cytoplasmic inclusions (Fig. 1); dystrophic neurites were less frequent [1, 11, 16, 17, 21, 26]. In contrast to poly-GP and poly-GA, we did not detect dystrophic neurites with immunohistochemistry for poly-GR. These results are consistent with a previous study that showed almost complete lack of poly-GR immunoreactive dystrophic neurites [26]. Glial cytoplasmic inclusions were also occasionally detected with poly-GA and poly-GP immunohistochemistry, but they were very rare with poly-GR immunohistochemistry.

### Poly-GR inclusion distribution among clinicopathologic subgroups

To evaluate whether poly-GA, poly-GP or poly-GR pathology differs among different clinicopathologic subgroups, we quantified lesion burden with image analysis. Although there were no significant differences between the subgroups for burden of poly-GA and poly-GP, poly-GR was significantly different in the three clinicopathologic subgroups. While overall lesions were sparse, we found greater burden of poly-GR inclusions in the FCtx of FTLD-MND compared to MND, and significantly more poly-GR inclusions in specific hippocampal subfields (i.e. CA2/3) of FTLD-MND compared to FTLD, as well as a trend for increases in CA4 of FTLD-MND compared to FTLD (Additional file 3: Table S1). We found poly-GR density was significantly different in FTD/MND in frontal cortex and hippocampus.

### Correlations between poly-GR pathology and neurodegeneration

**Fig. 2:**
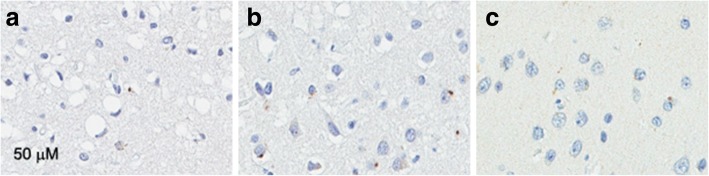
poly-GR density and neurodegenerationpoly-GR density is associated with neurodegeneration in FCtx and DF. Representative images show neurodegeneration and poly-GR inclusions. Most severe neurodegeneration with spongiosis and gliosis, but sparse poly-GR inclusions in FTLD (**a**) Severe neurodegeneration with spongiosis and gliosis in FTLD-MND (**b**) Mild neurodegeneration in MND (**c**). Scale bar represents 50 μM

We hypothesized that if poly-GR is neurotoxic, the density of poly-GR inclusions might be associated with neurodegeneration. We thus analyzed the burden of poly-GA, poly-GP and poly-GR, as well as pTDP-43 using a color deconvolution algorithm of digital images of regions of interest. In the same regions we scored the severity of neurodegeneration with semiquantitative methods. As expected, there were strong correlations between density of pTDP-43 pathology and neurodegeneration in FCtx (*r* = 0.46, *p* = 0.004), hippocampal DF (*r* = 0.40, *p* = 0.01) and CA4 (*r* = 0.61, *p* = 0.00006) (see Additional file 4: Figure S3). Interestingly, we also found a strong correlation between poly-GR density and neurodegeneration in FCtx (*r* = 0.45, *p* = 0.004), but a less robust correlation between poly-GR density and neurodegeneration in the hippocampal DF (*r* = 0.33, *p* = 0.04) (Table 2). Representative images of poly-GR and neurodegeneration are illustrated in Fig. 2. In contrast to other sense-strand DPR (poly-GA and poly-GP), poly-GR showed correlation with neurodegeneration even though the density of lesions was far less for poly-GR compared with poly-GA and poly-GP.

**Table 2:** Correlations of phospho-TDP-43 and DPR burden with neurodegeneration

	FCtx	DF	CA4
poly-GA	*r* = − 0.19	*r* = 0.20	*r* = 0.10
	*p* = 0.28	*p* = 0.24	*p* = 0.57
poly-GP	*r* = − 0.14	*r* = 0.26	*r* = 0.31
	*p* = 0.42	*p* = 0.12	*p* = 0.06
poly-GR	*r* = 0.45	*r* = 0.33	*r* = − 0.01
	*p* = 0.004	*p* = 0.04	*p* = 0.96
pTDP-43	*r* = 0.46	*r* = 0.40	*r* = 0.61
	*p* = 0.004	*p* = 0.013	*p* = 0.00006

P-values are from Pearson’s test of correlation. Significant *p*-values (< 0.05)

*FCtx* frontal cortex, *DF* dentate fascia, *CA4 cornu ammonis* sector 4

### Spectrum and distribution of poly-GR pathology and aDMA distribution

To assess our hypothesis that DMA modification may contribute to a possible toxic gain of function in poly-GR inclusions, we studied the morphology and distribution of inclusions immunoreactive for aDMA. We also quantified aDMA using western blot analysis in a subset of cases included in the larger immunohistochemical study. Blots were also probed with antibody to sDMA. The focus of this sub-analysis was on four FTLD-MND patients that had the most significant poly-GR pathology. Four FTLD-MND patients without *C9ORF72* mutations were included for comparison. We detected high molecular weight aDMA-immunoreactive species in brain lysates of c9FTLD-MND (Additional file 5: Figure S4), but not of sporadic FTLD-MND. We did not detect any differences between c9FTLD-MND and sporadic FTLD-MND for sDMA (Additional file 5: Figure S4).

Given these results, we focused only on aDMA in subsequent histopathologic studies. We characterized the spectrum of poly-GR and aDMA pathology in *C9ORF72* patients. As mentioned above, poly-GR neuronal inclusions were observed throughout the brain. Only a few glial inclusions and isolated neuronal intranuclear inclusions were detected, while no dystrophic neurites were detected (Fig. 1). Immunohistochemistry for aDMA labeled neuronal inclusions as well as many nuclei, the latter probably related to physiologic methylation of nuclear proteins such as histones (Fig. 1). As we observed for poly-GR, most of the inclusions immunoreactive for aDMA were neuronal cytoplasmic inclusions; glial inclusions were sparse, and no dystrophic neurites were found.

### Similar distribution of poly-GR and aDMA immunoreactivities in c9FTLD-MND brains

**Fig. 3:**
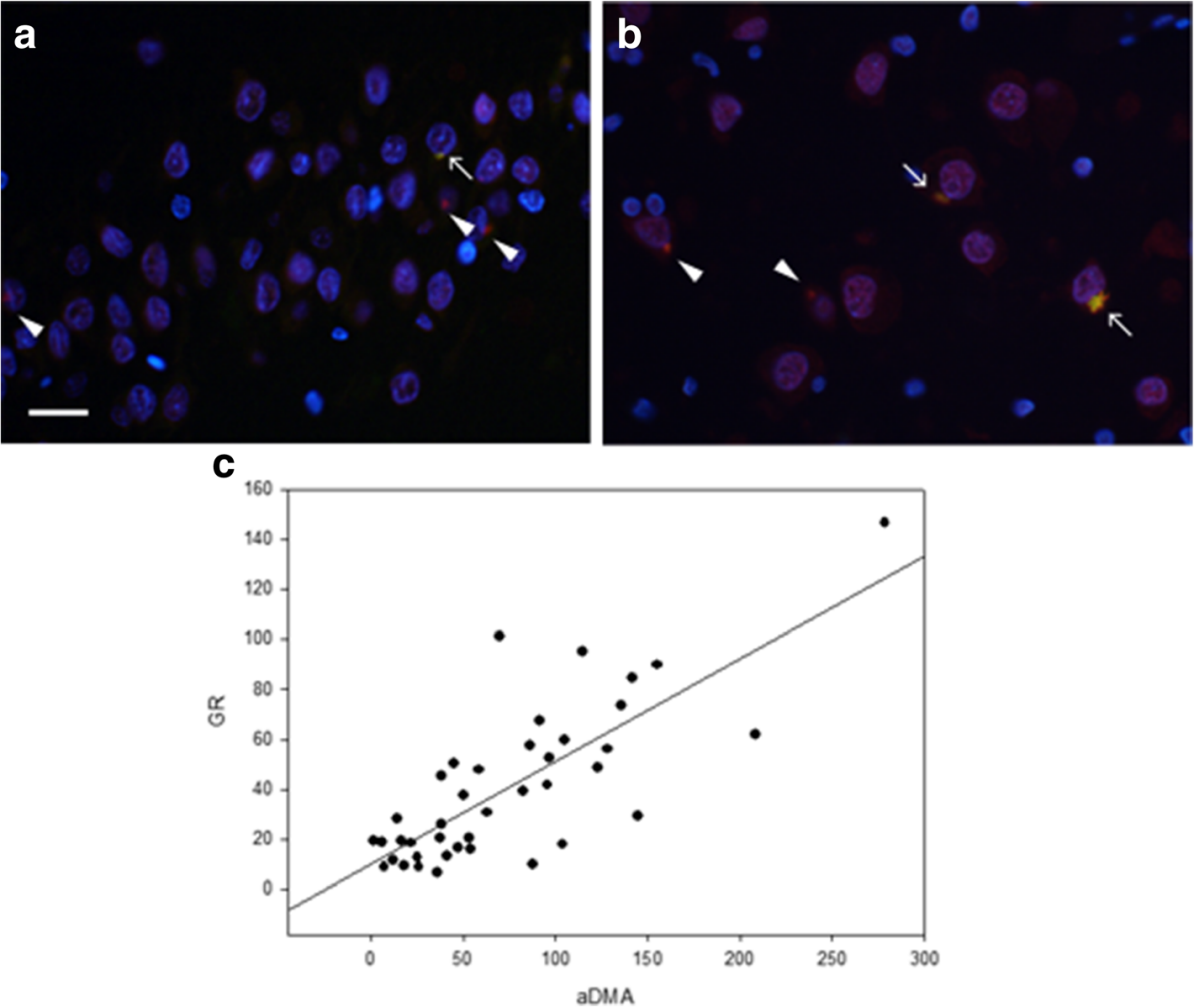
Colocalization of poly-GR and aDMA. Poly-GR neuronal cytoplasmic inclusions in the dentate fascia and CA4 of the hippocampus. Sparse poly-GR inclusions in the dentate fascia show colocalization with aDMA (arrow). Note that not all poly-GR aggregates contain aDMA (arrowheads) (**a**). Moderate poly-GR inclusions show colocalization with aDMA in CA4 (arrows). Again, not all poly-GR aggregates contain aDMA (arrowheads) (**b**). Scale bars: 10 μm. Plot shows the association of poly-GR and aDMA neuronal inclusions in the dentate fascia. The line shows linear regression (*r* = 0.77) (**c**)

**Fig. 4:**
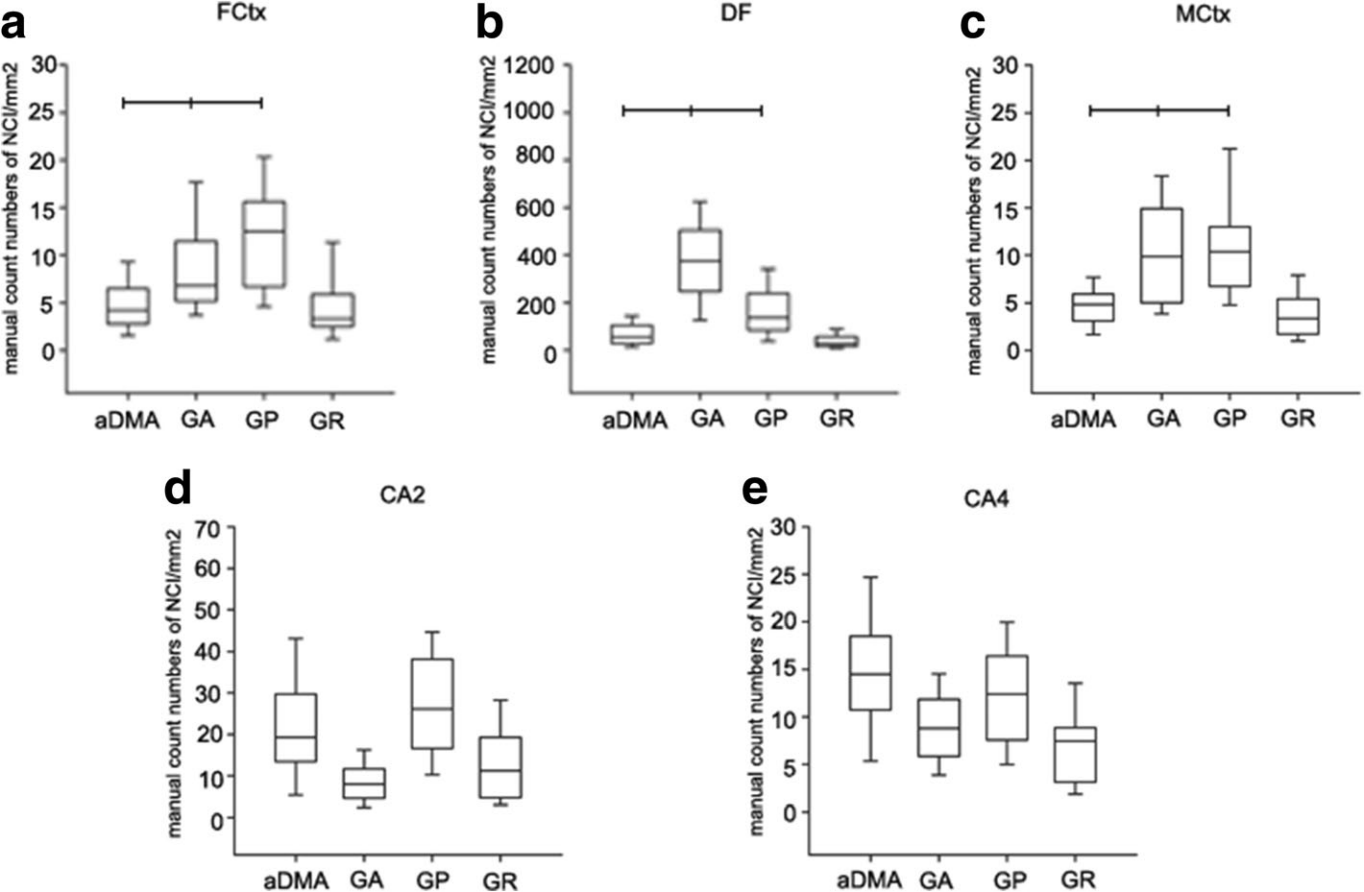
Overall frequency of inclusions for sense strand DPR and aDMA. Quantitative analysis of poly-GA, poly-GR and aDMA density in frontal cortex, hippocampus and motor cortex. The total number of inclusions counted in each case and the density of inclusions were calculated by total numbers of inclusions/total stained area (mm^2^). Frontal cortex (**a**), dentate fascia (**b**), motor cortex (**c**), CA2/3 (**d**), and CA4 (**e**). All variables analyzed with Kruskal-Wallis ANOVA on Ranks followed by Dunn’s post hoc test and data are displayed as median (25th and 75th range). *Statistically significant *p*-value (*p* < 0.05); all p-value for ANOVA on Ranks comparison of all groups

We used double immunofluorescence labeling to determine what proportion of the poly-GR inclusions also had aDMA immunoreactivity. We found frequent colocalization of poly-GR and aDMA in neuronal inclusions of the DF and CA4 sector of the hippocampus (Fig. 3). We performed manual counts of poly-GR and aDMA immunoreactive inclusions because excessive nuclear signal of aDMA precluded use of image analysis methods. We foun that manual counts and image analysis results were highly correlated (Additional file 2: Figure S2**)**. We found similar densities and distributions of poly-GR and aDMA immunoreactive inclusions in FCtx, MCtx and hippocampal DF (Fig. 4a-c). As expected, there was a strong correlation between poly-GR and aDMA (Fig. 3) and this was true for all brain regions analyzed (Additional file 6: Table S2). Taken together, these data suggest that a significant portion of poly-GR pathology may be methylated in *C9ORF72*-related disease.

### Clinicopathological correlations of poly-GR and aDMA pathology based on manual inclusion counts

We investigated poly-GA, poly-GP, poly-GR and aDMA immunoreactive inclusions in clinicopathologic subgroups, as assessed from manual counts. Similar to results described above, the density of poly-GR and aDMA immunoreactive neuronal inclusions was greater in patients with FTLD-MND compared to FTLD or MND. In the hippocampus, density of poly-GR inclusions was significantly greater in the DF, CA4 and CA2/3 of FTLD-MND compared to FTLD (Table 3). In addition, the density of aDMA inclusions was significantly greater in CA4 of FTLD-MND compared to FTLD. In frontal cortex, the density of poly-GR inclusions in FTLD-MND was significantly greater than in both FTLD and MND (Table 3). These results were similar to those obtained by image analysis using color deconvolution algorithms (Additional file 3: Table S1).

**Table 3:** Burden of DPR and aDMA by region in clinicopathologic subgroups

	FCtx	DF	CA4	CA2/3	MCtx
	poly-GA				
FTLD	6.4 (3.8, 9.4)	310 (110, 470)	6 (4.1, 9.5)	5.6 (4.5, 8.3)	5.8 (3.7, 12)
FTLD-MND	7.5 (5.6, 15)	420 (320, 630)	9.5 (6.6, 13)	9.7 (4.7, 15)	14 (6.2, 17)
MND	7.4 (5.8, 13)	380 (280, 480)	10 (7.4, 14)	8.2 (3.0, 12)	9.3 (6.5, 13)
	poly-GP				
FTLD	9.7 (5.9,14)	120 (71, 262)	8 (6.7, 13)	19 (13, 33)	10 (6.7, 13)
FTLD-MND	13 (11, 18)	180 (98, 270)	13 (7.4, 18)	31 (19, 39)	10 (8.0, 14)
MND	10 (6.6, 16)	130 (86, 230)	13 (10, 17)	27 (16, 39)	11 (4.9, 18)
	poly-GR				
FTLD	3.0 (2.2, 4.2)	16 (9.5, 48)	3.0 (2.3, 5.6)	5.1 (3.3, 9.3)	2.6 (1.3, 5.5)
FTLD-MND	6.8 (3.7, 9.5)^*^	49 (26, 86)^*^	8.5 (6.8, 12)^*^	17 (11, 26)^*^	4.7 (3.1, 7.9)
MND	2.9(1.1, 3.4)	26 (18, 51)	7.5 (4.7, 8.8)	12 (3.7, 22)	2.6 (1.2, 4.9)
	aDMA				
FTLD	3.3 (2.8, 4.7)	83 (31, 100)	10 (6.8, 14)	16 (10, 37)	3.4 (2.1, 5.6)
FTLD-MND	5.5 (3.1, 8.2)	77 (21, 140)	17 (14, 22)^*^	25 (17, 36)	5.6 (4.6, 6.8)
MND	4.0 (2.1, 7.4)	53 (31, 81)	15 (12, 20)	15 (6, 29)	3.5(2.5, 5.6)

In frontal cortex, *p* < 0.001, FTLD vs. FTLD-MND, *p* = 0.005, FTLD-MND v.s MND. In DF, *p* = 0.02, FTLD vs. FTLD-MND. In CA4, *p* = 0.002, FTLD vs. FTLD-MND. In CA2/3, *p* = 0.003, FTLD vs. FTLD-MND. All variables analyzed with Kruskal-Wallis ANOVA on Ranks and data are displayed as median (25th and 75th range)

^*^Statistically significant p-value (p < 0.05); all p-value for ANOVA on Ranks comparison of all three groups

*FCtx* frontal cortex, *MCtx* Motor cortex, *DF* dentate fascia, *CA* (*cornu ammonis*)

### In vitro evidence of poly-GR methylation

**Fig. 5:**
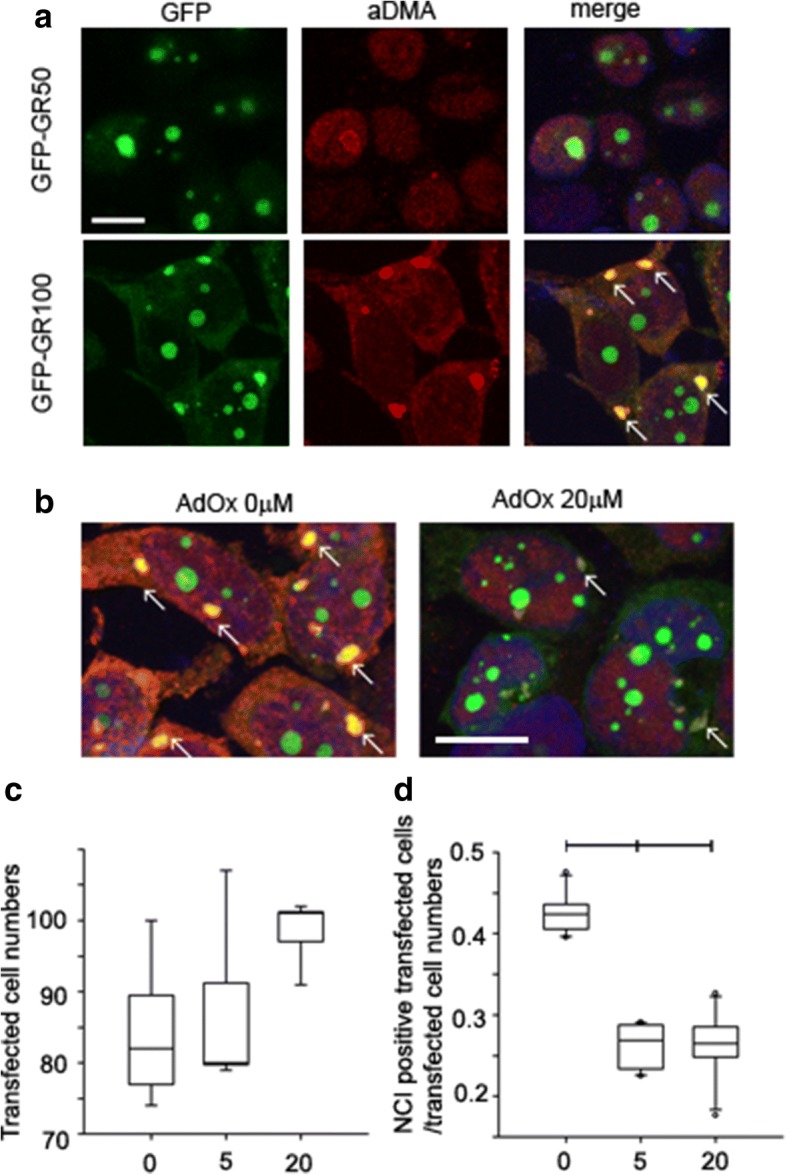
Poly-GR proteins form cytoplasmic inclusions in HEK-293 T cultured cells. **a** Expression of GFP-(GR)_50_ in HEK-293 T cells accumulate in nucleus. No cytoplasmic inclusions are detected. Expression of GFP-(GR)_100_ in HEK-293 T cells show immunoreactivity in nucleus and cytoplasm, with formation of cytoplasmic inclusions. Double immunofluorescence of GFP-(GR)_100_ and aDMA in HEK-293 T cells. **b** GFP-(GR)_100_ and aDMA. GFP-(GR)_100_ and no arginine methyltransferase inhibitor (AdOx) (left) versus AdOx 20 μM (right). Arrows indicate poly-GR and aDMA positive cytoplasmic aggregation (**c, d**) Effects of AdOx on poly-GR100 cytoplasmic inclusion formation. AdOx did not significantly decrease GFP positive, transfected cell numbers. **c** AdOx significantly decreased ratio of cytoplasmic inclusions in transfected cells – 43% at 0 mM, 27% at 5 mM and 25% at 20 mM. **d** Quantitative analysis and representative image showing cytoplasmic inclusions at different concentration of AdOx. Counts were made of over 500 transfected cells for each condition (with at least duplicate counts performed for each field analyzed). All variables analyzed with Kruskal-Wallis ANOVA on Ranks followed by Tukey’s post hoc analysis and data are displayed as median (25th and 75th range). *Statistically significant *p*-value (*p* < 0.05); all *p*-values for ANOVA on Ranks comparison of all three groups. Scale bars in (**a**) and (**b**): 20 μm

**Fig. 6:**
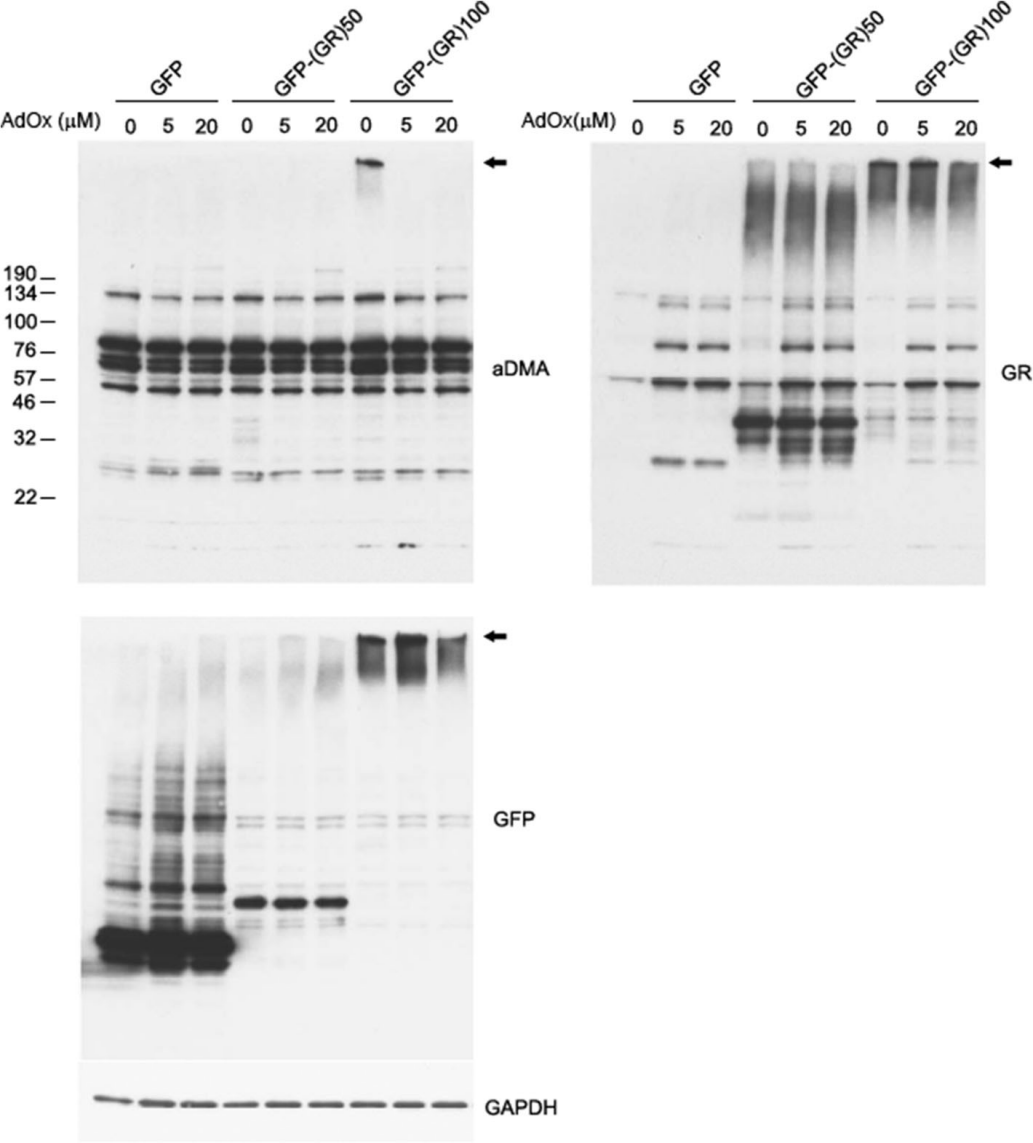
Western blot analysis of soluble and insoluble cell lysates. Immunoblots show poly-GR in high molecular weight species. Arrows mark the top of the gel. Only GFP-(GR)_100_ cells had dimethylarginine in high molecular species, which was lost after treatment with AdOx at 5- μM and 20-μM. *p < 0.05. ANOVA All p-value for ANOVA on ranks comparison of all three groups. Mann-Whitney U test was employed for the statistical analysis

To obtain further insight into poly-GR pathology in c9FTLD-MND, we overexpressed GFP-tagged poly-(GR)_50_ or poly-(GR)_100_ and treated the cells with adenosine dialdehyde (AdOx), a global methyltransferase inhibitor, at two concentrations (5 μM and 20 μM). GFP-(GR)_50_ accumulated only in the nucleus, whereas GFP-(GR)_100_ was detected in both the nucleus and cytoplasm. The cytoplasmic aggregates resembled inclusion bodies (Fig. 5). These results suggest that formation of poly-GR aggregates might be determined, in part, by repeat length, with longer repeats forming cytoplasmic inclusions resembling those seen in human brain cells (Fig. 1). To study the relationship between poly-GR aggregation and aDMA modification, we performed double immunofluorescent staining. There was frequent colocalization of poly-GR and aDMA in cytoplasmic aggregates of GFP-(GR)_100_ cells. Treating cells with AdOx decreased cytoplasmic aggregates in GFP-(GR)_100_ cells, while having no effect on the number of cells expressing GFP (Fig. 5c and d). To confirm immunofluorescent findings, we performed western blot analysis. Consistent with the results of immunostaining, only GFP-(GR)_100_ cells showed evidence of high molecular weight aDMA-immunoreactive species (arrows in Fig. 6). These results suggest that poly-GR aggregation may be modulated by arginine methylation.

## Discussion

An expanded hexanucleotide repeat in *C9ORF72* is the most frequent cause of FTLD and MND. One consequence of the expanded repeats is formation of DPR polymers through unconventional RAN translation [1, 21]. Despite increasing evidence from animal and cell culture models suggesting individual DPR have different degrees of toxicity, the manifestations of this toxicity and pathomechanisms are poorly understood in the human brain. Moreover, the relationship of DPR-related neuropathology with various clinicopathologic subtypes of *C9ORF72*-related disease is not well understood. In this study, we focused on poly-GR and provide evidence that density of poly-GR inclusions correlates with neurodegeneration more robustly than other DPR, especially in individuals with the most severe clinicopathologic phenotype, FTLD-MND. In addition, we explored a possible association of poly-GR toxicity with post-translational modification by methylation. In clinicopathological analysis, we demonstrated differences in distribution of poly-GR inclusions compared with poly-GA and found that certain brain regions, such as the hippocampal DF, have abundant poly-GA inclusions, but very few poly-GR inclusions. Moreover, poly-GR inclusions are closely associated with histologic features of neurodegeneration, especially in the hippocampus and FCtx. We found a similar distribution of poly-GR and aDMA-positive inclusions, and co-localization of poly-GR with aDMA. Of the three clinicopathologic phenotypes of *C9ORF72*-related disease, the FTLD-MND subgroup had the most inclusions immunoreactive for poly-GR and aDMA, especially in the FCtx and in several subregions of the hippocampus.

Only a few studies have addressed possible differences in DPR species in clinicopathological subtypes of *C9ORF72*-related disease. A semiquantitative neuropathologic study by Mackenzie and coworkers of 8 cases of FTLD, 16 of FTLD-MND and 11 of MND, found no relationship between specific DPR species and clinicopathologic phenotype, except for moderate association of poly-GA-positive dystrophic neurites with histologic markers of degeneration in the frontal cortex [17]. In another semiquantitative study by Schludi and coworkers in a cohort of 3 FTLD, 4 FTLD-MND and 2 MND, poly-PR inclusions were more abundant in FTLD than MND in the hippocampus, while poly-GA inclusions were more abundant in cerebellar granular layer of FTLD than both FTLD-MND and MND cases, but neither poly-PR nor poly-GA correlated with neurodegeneration [26]. In contrast, we found correlations between poly-GR and neurodegeneration in a larger series of cases using quantitative digital microscopic methods. Another unique finding in our study was that patients with the most severe clinicopathologic phenotype, namely those with both FTLD and MND, had higher lesion burden that the other two clinicopathologic groups. Like Schludi and coworkers [26], we found the highest density of poly-GR in FTLD-MND. On the other hand, we were not able to detect any difference between FTD and MND. The FTD group had the oldest age at death and longest disease duration; therefore, greater neurodegeneration might be associated with clearance of poly-GR pathology. While the three largest neuropathologic studies of DPR pathology in *C9ORF72*-related disease and a range of clinicopathologic subtypes, different quantitative methods and different antibodies for immunohistochemistry, there were several notable similarities. All found that poly-GR inclusions were less abundant than poly-GA (and poly-GP) inclusions and that certain brain regions had propensity for DPR inclusions, such as specific hippocampal subregions (DF, CA4 and CA2/3) and the cerebellar internal granular cell layer. The current study is the first to use digital pathology and quantitative methods, including color deconvolution image analysis, to objectively assess the burden of DPR pathology and histologic markers of neurodegeneration.

A recent study by Saberi and coworkers suggested poly-GR inclusions co-localize with TDP-43 in dendritic structures in a small series (*n* = 5) of MND compared with sporadic ALS (*n* = 3) [25]. These findings differ from our results and those of others [17, 26], who find little evidence for poly-GR immunoreactive dystrophic neurites.

Various pathomechanisms have been proposed for *C9ORF72*-related disease based on animal and cell culture models, including loss of function of *C9ORF72* leading to neuroinflammation [23], nuclear dysfunction due to alternative RNA splicing, perturbations in RNA metabolism, formation of DNA/RNA hybrids, RNA foci and defects in nucleocytoplasmic transport. The most characteristic pathologic hallmark of *C9ORF72*-related is widespread and regionally-specific neuronal DPR polymer inclusions and RNA foci. Given that two of the DPR contain arginine polymers, we hypothesized that post-translational modification of these DPR by methylarginine transferases might uniquely contribute to their greater toxicity. We found evidence to support methylation of poly-GR, while poly-PR was not amenable to study due to the paucity of these inclusions and the lack of reliable and sensitive reagents for their detection. Double immunostaining methods showed colocalization of aDMA immunoreactivity in poly-GR inclusions (Fig. 3). In contrast, there was very little colocalization of poly-GA and aDMA in neuronal inclusions (data not shown). Moreover, the distribution of poly-GR was highly correlated with the distribution of aDMA (Additional file 6: Table S2). We found that aDMA immunoreactivity was present in normal nuclei of various cell types, which is assumed to be physiologic methylation of nuclear proteins, such as histones and RNA-binding proteins [10]. Boeynaems and coworkers previously reported nuclear signal of aDMA in dentate fascia neurons of healthy controls [3]. We also detected nuclear aDMA in control cases (Additional file 7: Figure S5). Therefore, it is reasonable the aDMA nuclear labeling represents physiological methylation. In these proteins, DMA posttranslational modification occurs at sites with a consensus glycine/arginine rich (GAR) motif, which may be mimicked by poly-GR. Recent studies show that in addition to GAR-domain class of proteins, other proteins can be targeted by arginine methyltransferases [10]. Based upon histologic features and morphology, the nuclear aDMA we detected was not only in intranuclear inclusions, but also in other nuclear structures. Unlike normal controls, in c9FTLD-MND aDMA was found in the neuronal cytoplasm and it co-localized with poly-GR, especially in the hippocampus.

Given the strong correlation between poly-GR and aDMA, their association with neurodegeneration, and their relative abundance in patients with the most severe clinicopathologic phenotype (FTLD-MND), we hypothesized that post-translational modification of poly-GR may be a novel mechanism of gain of toxicity of poly-GR. In support of this, poly-GR cytoplasmic inclusions in cultured cells expressing GFP-(GR)_100_ were frequently co-labeled with a sensitive and specific antibody to aDMA. Treating the cells with an arginine methyltransferase inhibitor (AdOx), which has intrinsic cytotoxic properties, decreased the numbers of poly-GR cytoplasmic inclusions at times and concentrations not associated with significant cytotoxicity. These findings suggest that formation of poly-GR cytoplasmic inclusions might be influenced by aDMA modification.

Although post-translational modification of proteins with aDMA is often toxic in various tissues in human diseases, including the brain [5, 27, 29], the effects of post-translational aDMA modification is not well understood in the brain. Regarding clinical and experimental studies of CNS disease and aDMA, in hepatic encephalopathy, elevated aDMA levels could potentially contribute to cognitive dysfunction through oxidative stress, restriction of cerebral blood flow and inflammation [5]. In ischemic stroke, elevated levels of aDMA might contribute to brain injury through endothelial cell damage, and aDMA also might be involved in nitric oxide associated oxidative stress and excitotoxicity [4]. In neurodegenerative disorders, Suarez-Calved and coworkers reported that mono-methylated arginine is frequent in FTLD-FUS, but not in ALS-FUS. Unmethylated and mono-methylated methylarginine FUS had much higher binding affinities to the nuclear receptor of FUS, transportin-1, compared to aDMA FUS [28]. To complicate matters, DMA modification is also considered to both positively and negatively regulate protein-protein interactions [10].

A limitation of our study is the inability to detect specific methylated forms (e.g., mono-methylation, symmetrical di-methylation and asymmetrical di-methylation) of poly-GR (and poly-PR). There is also no direct way to know the effect of the DPR polymer repeat length on post-translational modification. Our in vitro cell culture studies used repeat lengths of 50- and 100-mers. These are much smaller repeats that seen in disease brain based upon their mobility on western blots. In this regard, it is interesting to note that Bennion Callister and co-workers pointed out that repeat length effects subcellular distribution of DPR, with smaller repeats producing inclusions in the nucleus (specifically in nucleoli) and larger repeats (> 1000) producing inclusions in the cytoplasm [2]. We were able to detect both nuclear and cytoplasmic inclusions in our cell culture models with much smaller repeats. It remains to be determined if repeat length affects methylation modification. Nevertheless, we suggest that current studies provide preliminary evidence from human neuropathology that post-translational modification of arginine residues may play a role in the toxicity of poly-GR. Clearly, there is need for further study.

## Conclusions

In summary, we provide evidence that poly-GR is more closely associated with markers of neurodegeneration than poly-GA and poly-GP. Preliminary evidence suggests that methylation may contribute to this toxicity. Furthermore, in our small clinicopathologic series poly-GR seems to be more abundant in patients with FTLD-MND compared to FTLD or MND. Investigating the contribution of poly-GR to neuronal dysfunction, TDP-43 pathology and neurodegeneration may shed additional light on disease pathogenesis and lead to new therapeutic targets.

## Additional files

Additional file 1: **Figure S1.** Semiquantitative assessment of neurodegeneration. Grading neurodegeneration in FCtx, hippocampal DF, CA4 and CA2/3 are shown. In FCtx, neurodegeneration was assessed by superficial microvacuolation and spongiosis. In DF, gaps in granular cell density were assessed as neurodegeneration. In CA4 and CA2/3, neuronal cell loss and gliosis were assessed as neurodegeneration. Neurodegeneration was graded as absent (G0), mild (G1), moderate (G2) or severe (G3). (TIF 1630 kb)

Additional file 2: **Figure S2.** Correlation between manual counts and positive pixel burden from color deconvolution in poly-GR staining. Plot shows the correlation of manual counts of neuronal cytoplasmic inclusions and positive pixel burden from color deconvolution in poly-GR staining. The line shows linear regression CD color deconvolution. Ctx frontal cortex, DF dentate fascia, CA - *cornu ammonis*, MCtx motor cortex. (TIF 2581 kb)

Additional file 3: **Table S1.** Quantitative assessment of DPR density by color deconvolution algorithm in clinicopathologic subgroups of C9ORF72-related disease. In frontal cortex, *p* = 0.019, FTLD vs. FTLD-MND. In CA4, *p* = 0.055, FTLD vs. FTLD-MND. In CA2/3, *p* = 0.03, FTLD vs. FTLD-MND. Significant *p*-values (< 0.05) are indicated in bold. All variables were analyzed with Kruskal-Wallis ANOVA on Ranks and data are displayed as median (25th and 75th range). *Statistically significant p-value (*p* < 0.05); all p-value for ANOVA on Ranks comparison of all three groups. FCtx = frontal cortex, MCtx = Motor cortex, DF dentate fascia, CA hippocampal subfields. (DOCX 15 kb)

Additional file 4: **Figure S3.** Dot plot graph of semiquantitative assessment of neurodegeneration and DPR. Note that X axis is neurodegeneration score (0 to 3), Y-axis is density of DPR. FCtx - frontal cortex, DF - dentate fascia, CA4 - *cornu ammonis* sector 4. (TIF 1325 kb)

Additional file 5: **Figure S4.** Western blot analysis of aDMA and sDMA in brains of C9FLTD-MND and sporadic FTLD-MND. The high molecular weight aDMA and sDMA signals are visible in c9FTD/ALS, but not in sporadic FTD/ALS cases. (TIF 2960 kb)

Additional file 6: **Table S2.** Correlation between DPR and aDMA inclusions. *P*-values are from Pearson’s test of correlation. Significant *p*-values (< 0.05) are indicated in bold. FCtx frontal cortex, DF dentate fascia, CA - *cornu ammonis*, MCtx motor cortex. (DOCX 14 kb)

Additional file 7: **Figure S5** Comparison of immunostaining with aDMA between C9ORF72 cases and non- neurodegeneration control in parahippocampal cortex. The nuclear signal of aDMA is variable in both cases and controls. Note sparse cytoplasmic inclusions labeled with aDMA in in C9ORF72 cases (arrows). Scale bar represents 50 μM. (TIF 2898 kb)
