# Supplementary figures and images for: Poly-GR dipeptide repeat polymers correlate with neurodegeneration and Clinicopathological subtypes in C9ORF72-related brain disease
Source: Acta Neuropathol Commun. 2018 Jul 20;6:63. doi: 10.1186/s40478-018-0564-7 (PMC6054740; doi:10.1186/s40478-018-0564-7)

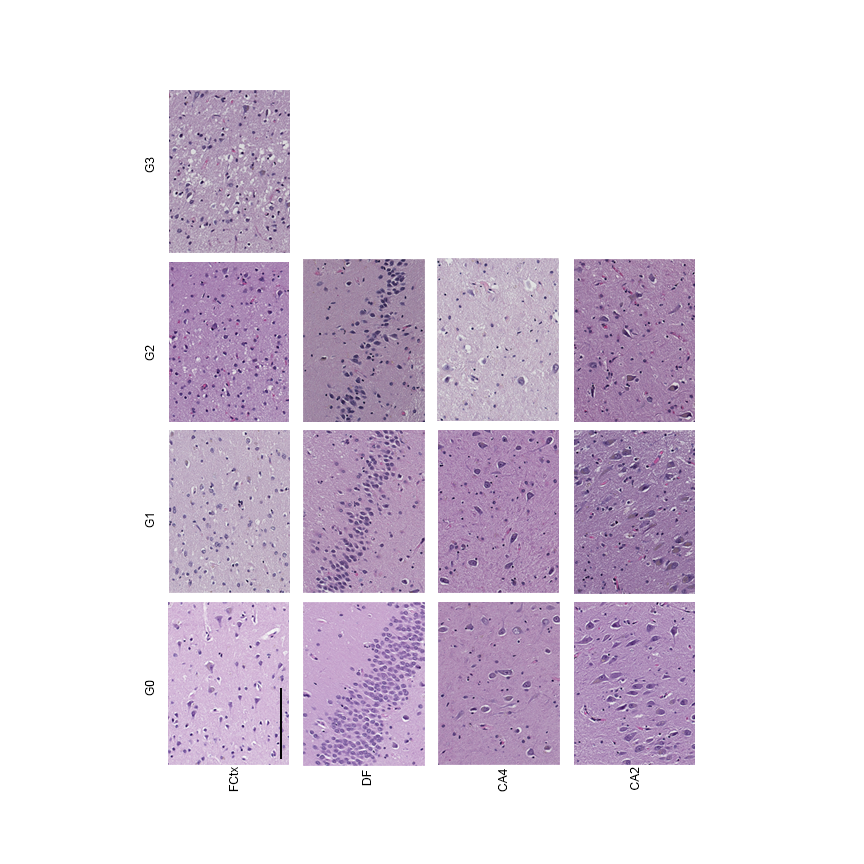

Supplement: Supplementary file 1 — Figure S1. Semiquantitative assessment of neurodegeneration. Grading neurodegeneration in FCtx, hippocampal DF, CA4 and CA2/3 are shown. In FCtx, neurodegeneration was assessed by superficial microvacuolation and spongiosis. In DF, gaps in granular cell density were assessed as neurodegeneration. In CA4 and CA2/3, neuronal cell loss and gliosis were assessed as neurodegeneration. Neurodegeneration was graded as absent (G0), mild (G1), moderate (G2) or severe (G3). (TIF 1630 kb) [file 40478_2018_564_MOESM1_ESM.tif]

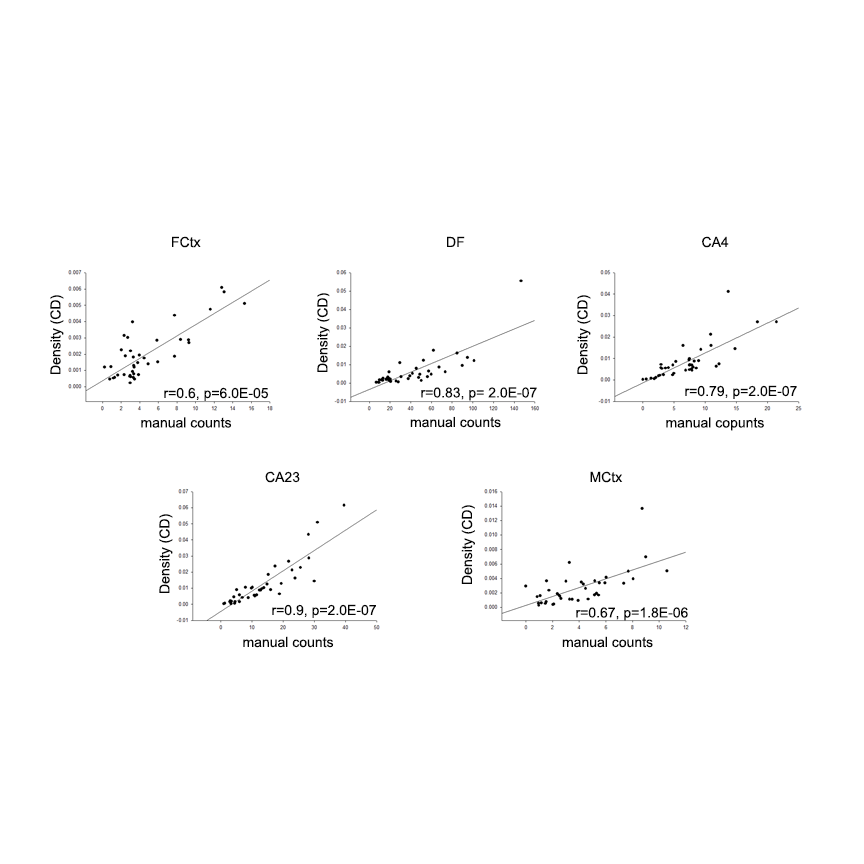

Supplement: Supplementary file 2 — Figure S2. Correlation between manual counts and positive pixel burden from color deconvolution in poly-GR staining. Plot shows the correlation of manual counts of neuronal cytoplasmic inclusions and positive pixel burden from color deconvolution in poly-GR staining. The line shows linear regression CD color deconvolution. Ctx frontal cortex, DF dentate fascia, CA - cornu ammonis, MCtx motor cortex. (TIF 2581 kb) [file 40478_2018_564_MOESM2_ESM.tif]

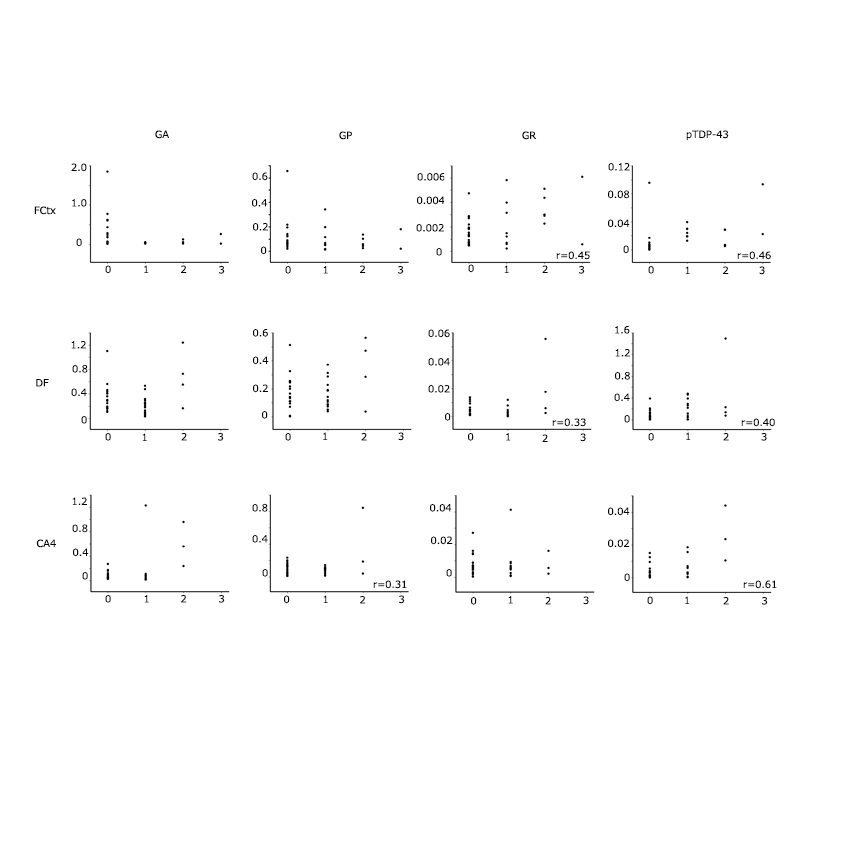

Supplement: Supplementary file 4 — Figure S3. Dot plot graph of semiquantitative assessment of neurodegeneration and DPR. Note that X axis is neurodegeneration score (0 to 3), Y-axis is density of DPR. FCtx - frontal cortex, DF - dentate fascia, CA4 - cornu ammonis sector 4. (TIF 1325 kb) [file 40478_2018_564_MOESM4_ESM.tif]

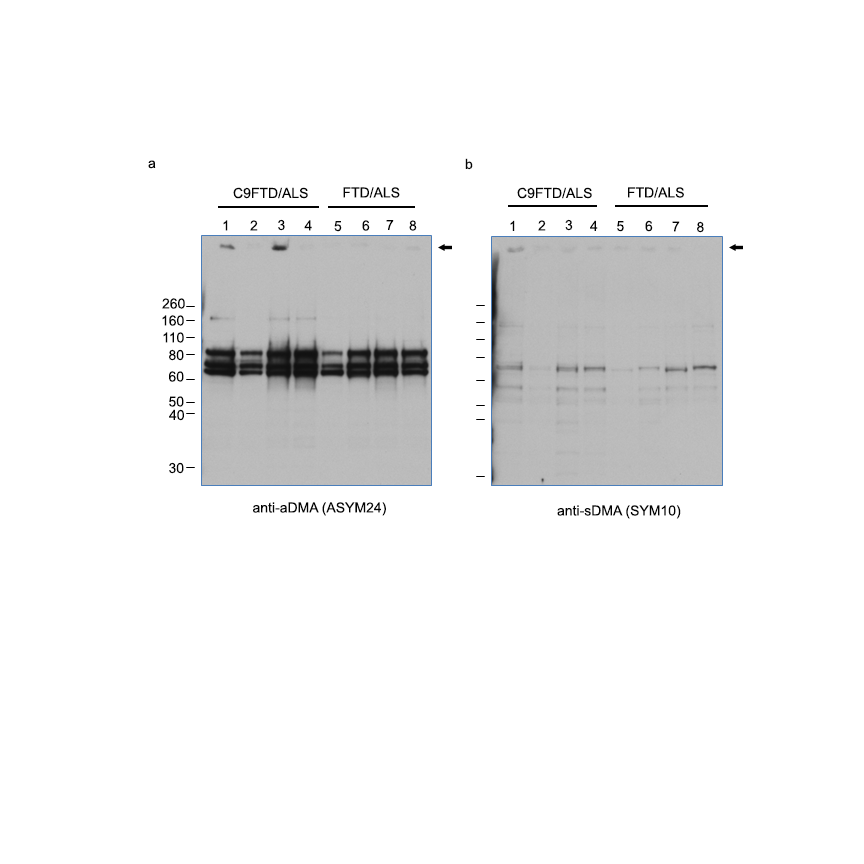

Supplement: Supplementary file 5 — Figure S4. Western blot analysis of aDMA and sDMA in brains of C9FLTD-MND and sporadic FTLD-MND. The high molecular weight aDMA and sDMA signals are visible in c9FTD/ALS, but not in sporadic FTD/ALS cases. (TIF 2960 kb) [file 40478_2018_564_MOESM5_ESM.tif]

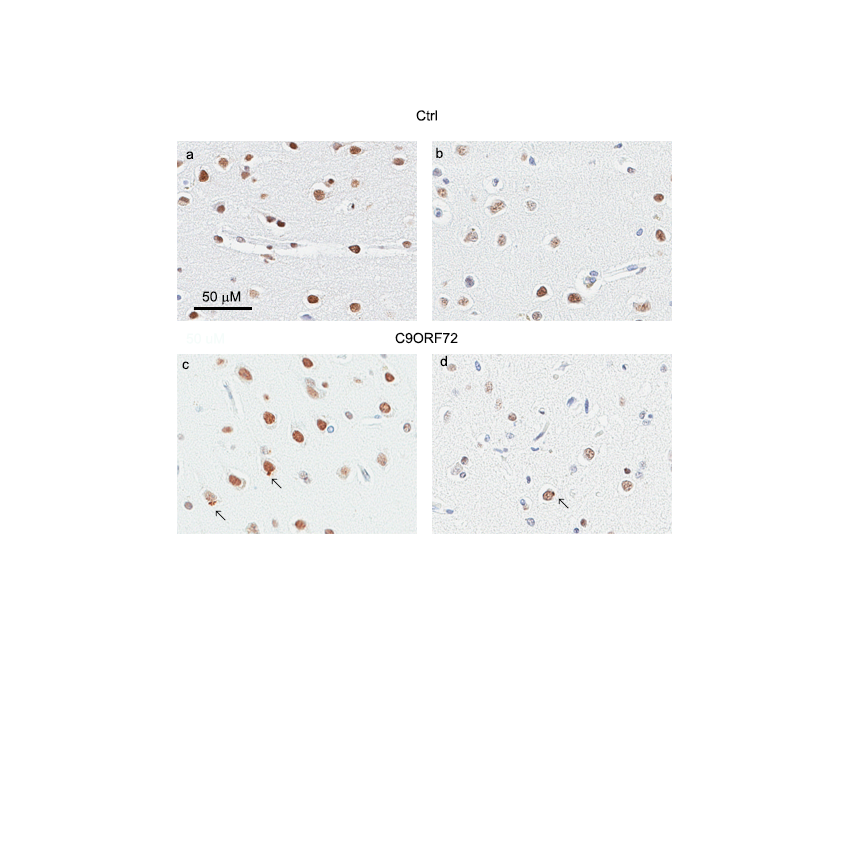

Supplement: Supplementary file 7 — Figure S5 Comparison of immunostaining with aDMA between C9ORF72 cases and non- neurodegeneration control in parahippocampal cortex. The nuclear signal of aDMA is variable in both cases and controls. Note sparse cytoplasmic inclusions labeled with aDMA in in C9ORF72 cases (arrows). Scale bar represents 50 μM. (TIF 2898 kb) [file 40478_2018_564_MOESM7_ESM.tif]
