# Supplementary material for: Poly-GR dipeptide repeat polymers correlate with neurodegeneration and Clinicopathological subtypes in C9ORF72-related brain disease
Source: Acta Neuropathol Commun. 2018 Jul 20;6:63. doi: 10.1186/s40478-018-0564-7 (PMC6054740; doi:10.1186/s40478-018-0564-7)
Supplement: Supplementary file 3 — Table S1. Quantitative assessment of DPR density by color deconvolution algorithm in clinicopathologic subgroups of C9ORF72-related disease. In frontal cortex, p = 0.019, FTLD vs. FTLD-MND. In CA4, p = 0.055, FTLD vs. FTLD-MND. In CA2/3, p = 0.03, FTLD vs. FTLD-MND. Significant p-values (< 0.05) are indicated in bold. All variables were analyzed with Kruskal-Wallis ANOVA on Ranks and data are displayed as median (25th and 75th range). *Statistically significant p-value (p < 0.05); all p-value for ANOVA on Ranks comparison of all three groups. FCtx = frontal cortex, MCtx = Motor cortex, DF dentate fascia, CA hippocampal subfields. (DOCX 15 kb) [file 40478_2018_564_MOESM3_ESM.docx]

**Table S1. Lesion burden for each DPR by clinicopathologic subgroup**

|  | **FTLD (n=13)** | **FTLD-MND (n=14)** | **MND (n=13)** | **p-value** |
| --- | --- | --- | --- | --- |
|  | **FCtx** | | |  |
| **GA** | 0.04 (0.02, 0.08) | 0.06 (0.03, 0.2) | 0.03 (0.02, 0.08) | 0.31 |
| **GP** | 0.06 (0.02,0.07) | 0.09(0.06,0.19) | 0.06(0.04,0.13) | 0.06 |
| **GR** | 0.001 (0.001, 0.003) | 0.002 (0.001, 0.004)* | 0.001 (0.001, 0.002) | 0.04 |
|  | **DF** | | |  |
| **GA** | 0.25 (0.12, 0.50) | 0.29 (0.24, 0.68) | 0.19(0.14, 0.35) | 0.19 |
| **GP** | 0.12 (0.07,0.25) | 0.18(0.10, 0.29) | 0.12(0.10,0.29) | 0.74 |
| **GR** | 0.003 (0.001, 0.005) | 0.004 (0.002, 0.01) | 0.002 (0.001, 0.007) | 0.32 |
|  | **CA4** | | |  |
| **GA** | 0.08 (0.04, 0.10) | 0.08 (0.05, 0.14) | 0.06(0.05, 0.09) | 0.71 |
| **GP** | 0.08(0.03, 0.11) | 0.13(0.03, 0.14) | 0.10(0.06,0.19) | 0.41 |
| **GR** | 0.005 (0.001, 0.007) | 0.007 (0.005, 0.01) | 0.007 (0.004, 0.01) | 0.14 |
|  | **CA2/3** | | |  |
| **GA** | 0.3 (0.05, 1.0) | 0.15(0.05, 0.87) | 0.04(0.02, 0.20) | 0.08 |
| **GP** | 0.10(0.06,0.24) | 0.14(0.09,0.23) | 0.13(0.08,0.33) | 0.72 |
| **GR** | 0.002 (0.002, 0.01) | 0.01 (0.006, 0.02)* | 0.009 (0.003, 0.014) | 0.03 |
|  | **MCtx** | | |  |
| **GA** | 0.05 (0.03, 0.14) | 0.16(0.02, 0.39) | 0.06(0.05, 0.08) | 0.37 |
| **GP** | 0.05 (0.04, 0.11) | 0.07(0.03, 0.19) | 0.10(0.06, 0.18) | 0.37 |
| **GR** | 0.002 (0.001, 0.004) | 0.003 (0.01, 0.004) | 0.002 (0.002, 0.003) | 0.45 |
