# Supplementary material for: Poly-GR dipeptide repeat polymers correlate with neurodegeneration and Clinicopathological subtypes in C9ORF72-related brain disease
Source: Acta Neuropathol Commun. 2018 Jul 20;6:63. doi: 10.1186/s40478-018-0564-7 (PMC6054740; doi:10.1186/s40478-018-0564-7)
Supplement: Supplementary file 6 — Table S2. Correlation between DPR and aDMA inclusions. P-values are from Pearson’s test of correlation. Significant p-values (< 0.05) are indicated in bold. FCtx frontal cortex, DF dentate fascia, CA - cornu ammonis, MCtx motor cortex. (DOCX 14 kb) [file 40478_2018_564_MOESM6_ESM.docx]

**Table S2. Correlations between burden of DPR and aDMA**

| **FCtx** | **DF** | **CA4** | **CA2/3** | **MCtx** |  |
| --- | --- | --- | --- | --- | --- |
|  |  | **GA** |  |  |  |
| r = 0.74 | r = 0.63 | r = 0.85 | r = 0.43 | r = 0.57 |  |
| p = 5.4 x E-08 | p = 1.4 x E-05 | p = 3.9 x E-12 | p = 5.3 x E-03 | p = 1.6 x E-04 |  |
|  |  | **GP** |  |  |  |
| r = 0.46 | r = 0.59 | r = 0.58 | r = 0.43 | r = 0.50 |  |
| p = 3.4 x E-03 | p = 6.8 x E-05 | p = 8.9 x E-05 | p = 5.9 x E-03 | p = 1.3 x E-03 |  |
|  |  | **GR** |  |  |  |
| r = 0.52 | r = 0.77 | r = 0.80 | r = 0.50 | r = 0.73 |  |
| p = 6.3 x E-04 | p = 4.6 x E-09 | p = 1.0 x E-09 | p = 9.7 x E-04 | p = 2.3 x E-07 |  |
